# Calorie Restriction Extends Life Span— But Which Calories?

**DOI:** 10.1371/journal.pmed.0020231

**Published:** 2005-08-30

**Authors:** Leonie K Heilbronn, Eric Ravussin

## Abstract

Heilbronn and Ravussin discuss the findings and implications of a study in *PLoS Biology* of caloric restriction in fruit flies. Are there implications for humans?

Evidence that calorie restriction (CR) retards aging and extends median and maximal life span was first described in the 1930s by McCay et al. [1]. Since then, similar observations have been made in a variety of species including rodents, fish, fruit flies, worms, and yeast [2]—and although they are not yet definitive, results from ongoing longevity studies in monkeys suggest that CR will also extend life span in longer-lived species [3,4].

There are many theories explaining the mechanisms by which CR extends life span. An early hypothesis was that delayed sexual maturation might be a mechanism. However, it has since been shown that CR initiated in older animals also increases life span [5]. Reduced metabolic rate—with consequent reduction in free radical production—was another early, leading hypothesis to explain the anti-aging effects of CR. But there are many other metabolic effects that have been associated with CR, including altered insulin sensitivity and signaling, stress resistance, altered neuroendocrine function, and changes in nutrient signaling. Any, or a combination, of these biological changes may retard aging. However, recent studies seem to favor a highly conserved stress response that evolved early in most species to increase an organism's chance of surviving adversity (such as CR) by triggering concerted physiological responses [6].

## Nutrient Composition of Calorie Restricted Diets

Previous studies in rodents have shown that the effects of CR on extending life span are dose dependent, with a 20% reduction in calorie intake producing a smaller increment in life span as compared to a 40% reduction in food intake [7]. From this work and others, the reduction in calories was recognized to be of paramount importance in the longevity response, and alterations in the nutrient content of diets were considered irrelevant. However, a recent study in *PLoS Biology* by Mair et al. challenges this long-held concept of CR [8].

## From Fruit Flies to Rodents

**Figure 1:**
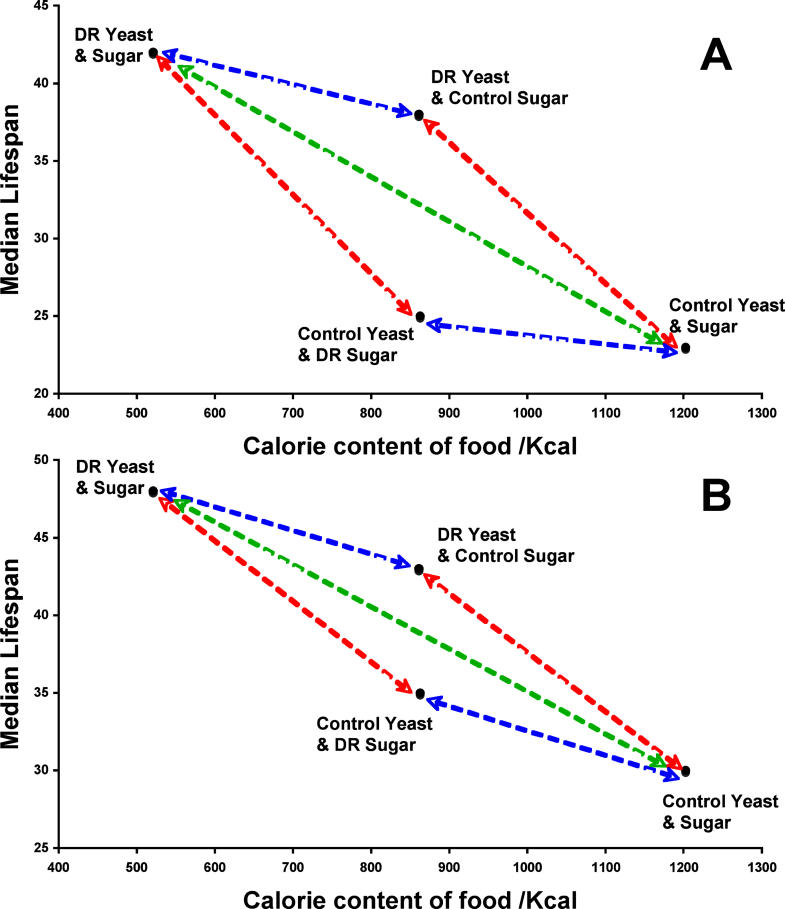
Plot of Median Life Span of Female Drosophila against the Estimated Caloric Content of the Food Medium (A) and (B) represent independent repeats. Red arrows link pairs of food types where differences in caloric content are due to different yeast concentrations. Blue arrows link pairs of food types where differences in caloric content are due to different sugar concentrations. Green arrow links food types where differences in caloric content are due to both different sugar and yeast concentrations. Life span is extended to a greater extent per calorie by reducing yeast concentration from control to DR levels than by reducing sugar. This is in contrast to what would be predicted if calorie intake were the key mediator of life-span extension by DR in fruit flies. (Figure from [8])

In the study by Mair et al., the authors examined life span in fruit flies (Drosophila melanogaster) fed one of four different diets: (1) a combination of yeast and sugar (control), (2) restricted in yeast only, (3) restricted in sugar only, and (4) restricted in yeast and sugar. The authors observed that in the restricted sugar group, as compared to the controls, maximal life span was unchanged and median life span was increased by only 12%. On the other hand, both maximal and median life spans were increased substantially in the restricted yeast group and in the restricted yeast and sugar group (Figure 1). Importantly, the authors claim that total calorie contents of the restricted sugar and restricted yeast diets were similar. Thus, from this study it can be implied that restricting carbohydrate is less advantageous than restricting protein/lipid for mediating the effects of dietary restriction (DR) on life span.

It is of concern that the authors did not directly measure the flies' total food intake but only estimated intake by examining their feeding behavior. This method may not take into account possible differences in the rate of food uptake of restricted flies, which could affect the results.

Investigators have undertaken studies of prolonged calorie restriction in humans

Only a handful of studies have investigated the role of altering nutrient composition on longevity in rodents. The results from these studies have been contradictory, with some studies showing no life-span extension following restriction of fat only [9] and others showing increased life span following replacement of casein-protein for soy-protein [10]. However, the concept that it is not just reduced calorie intake that drives the life-span extension effect is not new, and the timing of food intake has also been proposed to be of importance. For example, when rodents are fed every other day, improvements in biomarkers of aging and increased life span are observed, even though measured calorie intake and body weight were not statistically different from ad-libitum or pair-fed animals [11,12]. These responses were dependent on genotype and the age at which the protocol was implemented. Furthermore, animals fed every other day had a better response to neurotoxic stressors as compared to animals maintained on prolonged CR.

The mechanisms behind the differences in restricting carbohydrate vs. protein/lipid on life-span extension were not examined in the Mair et al. study [8]. Such mechanisms obviously imply the existence of molecular systems in cells that sense macronutrients—systems that may respond not only to nutrient availability but also to the hormonal response elicited by these dietary nutrients [13]. For example, restricting dietary carbohydrates increases the plasma concentration of B-hydroxybutyrate (that is, ketogenesis), a shift that may counteract life-span extension in mammals. However, rat models of Alzheimer and Parkinson diseases fed a ketogenic diet exhibit increased resistance to seizures and have increased protection of neurons [14]. Ketogenic diets are also prescribed to patients with epilepsy, and although there have been no randomized controlled trials, large observational studies (some prospective) suggest that this diet does have a beneficial effect on seizures [15].

However, it is likely that carbohydrate and protein may differentially alter nutrient-sensing pathways such as Sir2 and mammalian target of rapomyocin (mTOR), which are gaining acceptance as mediators of the life-span extension effects of CR [16]. Sir2 (the mammalian homolog is SIRT1) is a nicotinamide-adenine-dinucleotide-dependent histone deacetylase that interacts with numerous transcription factors to silence gene transcription. Sir2 is upregulated by CR and is required for life-span extension effects of CR in Caenorhabditis elegans (reviewed in [6]). mTOR is a serine/threonine kinase that is activated by insulin, nutrients, and growth factors and is a central regulator of ribosome biogenesis, protein synthesis, and cell growth. Inhibition of mTOR increases life span in Drosophila and C. elegans (reviewed in [16]).

## Are There Implications for Human Life Span?

Obviously, invertebrate organisms cannot serve as reliable models for human longevity, and the results by Mair et al. [8] should not be extrapolated to mammals in general. But if this result could be replicated in humans, then the prospect of DR to increase life span would be eminently more attractive than overall CR. This would mean that a change in food patterns could have a similar effect to the dramatic reduction of total food intake. However, the life-span extension effects of CR have not been proven in humans, and the jury is still out on whether nutrient composition will even affect life span in non-human primates.

In close collaboration with the National Institute on Aging, investigators in Baton Rouge, Boston, and Saint Louis (all in the United States) have undertaken studies of prolonged CR in humans. These studies aim to test the feasibility and safety of different types of calorie restricted diets in non-obese people and to determine the effects of CR on risk factors for age-related diseases, psychological factors, immune function, oxidative stress, and molecular pathways identified in lower species [17]. These kinds of studies will further help identify the mechanisms underpinning the effect of CR or DR on longevity.

## Abbreviations

CR: calorie restriction
DR: dietary restriction
mTOR: mammalian target of rapomyocin

## References

[pmed-0020231-b1] McCay CM, Crowel MF, Maynard LA (1935). The effect of retarded growth upon the length of the life span and upon the ultimate body size. J Nutr.

[pmed-0020231-b2] Weindruch R, Walford RL (1988). The retardation of aging and disease by dietary restriction.

[pmed-0020231-b3] Bodkin NL, Alexander TM, Ortmeyer HK, Johnson E, Hansen BC (2003). Mortality and morbidity in laboratory-maintained Rhesus monkeys and effects of long-term dietary restriction. J Gerontol A Biol Sci Med Sci.

[pmed-0020231-b4] Lane MA, Black A, Ingram DK, Roth GS (1998). Calorie restriction in non-human primates: implications for age-related disease risk. J Anti-Aging Med.

[pmed-0020231-b5] Weindruch R, Walford RL (1982). Dietary restriction in mice beginning at 1 year of age: Effect on life-span and spontaneous cancer incidence. Science.

[pmed-0020231-b6] Sinclair DA (2005). Toward a unified theory of caloric restriction and longevity regulation. Mech Ageing Dev.

[pmed-0020231-b7] Weindruch R, Walford RL, Fligiel S, Guthrie D (1986). The retardation of aging in mice by dietary restriction: Longevity, cancer, immunity and lifetime energy intake. J Nutr.

[pmed-0020231-b8] Mair W, Piper MDW, Partridge L (2005). Calories do not explain extension of life span by dietary restriction in Drosophila. PLoS Biol.

[pmed-0020231-b9] Iwasaki K, Gleiser CA, Masoro EJ, McMahan CA, Seo EJ (1988). Influence of the restriction of individual dietary components on longevity and age-related disease of Fischer rats: The fat component and the mineral component. J Gerontol.

[pmed-0020231-b10] Iwasaki K, Gleiser CA, Masoro EJ, McMahan CA, Seo EJ (1988). The influence of dietary protein source on longevity and age-related disease processes of Fischer rats. J Gerontol.

[pmed-0020231-b11] Anson RM, Guo Z, de Cabo R, Iyun T, Rios M (2003). Intermittent fasting dissociates beneficial effects of dietary restriction on glucose metabolism and neuronal resistance to injury from calorie intake. Proc Natl Acad Sci U S A.

[pmed-0020231-b12] Goodrick CL, Ingram DK, Reynolds MA, Freeman JR, Cider N (1990). Effects of intermittent feeding upon body weight and life span in inbred mice: Interaction of genotype and age. Mech Ageing Dev.

[pmed-0020231-b13] Rodgers JT, Lerin C, Haas W, Gygi SP, Spiegelman BM (2005). Nutrient control of glucose homeostasis through a complex of PGC-1[alpha] and SIRT1. Nature.

[pmed-0020231-b14] Kashiwaya Y, Takeshima T, Mori N, Nakashima K, Clarke K (2000). D-beta-Hydroxybutyrate protects neurons in models of Alzheimer's and Parkinson's disease. Proc Natl Acad Sci U S A.

[pmed-0020231-b15] Levy R, Cooper P (2003). Ketogenic diet for epilepsy. Cochrane Database Syst Rev.

[pmed-0020231-b16] Beckman M (2004). More without TOR. Inhibiting nutrient sensor extends life span in fruit flies. Sci Aging Knowledge Environ.

[pmed-0020231-b17] Heilbronn LK, Ravussin E (2003). Calorie restriction and aging: review of the literature and implications for studies in humans. Am J Clin Nutr.

